# The Role of Plant-Derived Compounds on the Cervical Spinal Cord After Sciatic Nerve Transection in Diabetic Rats

**DOI:** 10.3390/ph19081301

**Published:** 2026-08-17

**Authors:** Abdullah Hilmi Marangoz, Burcu Delibaş, Gamze Altun, Arife Ahsen Kaplan, Hala Mahgoub Hamour, Abit Aktaş, Sait Polat, Süleyman Kaplan

**Affiliations:** 1 Department of Neurosurgery, Faculty of Medicine, Ondokuz Mayıs University, 55139 Samsun, Türkiye; 2 Department of Histology and Embryology, Faculty of Medicine, Recep Tayyip Erdoğan University, 53200 Rize, Türkiye; burcu.delibas@erdogan.edu.tr; 3 Department of Histology and Embryology, Faculty of Medicine, Ondokuz Mayıs University, 55139 Samsun, Türkiye; gamze.altun@omu.edu.tr (G.A.); dr.hala.hamour@gmail.com (H.M.H.); skaplanomu@yahoo.com (S.K.); 4 Department of Histology and Embryology, Faculty of Medicine, Bolu Abant İzzet Baysal University, 14030 Bolu, Türkiye; ahsenkaplan@gmail.com; 5 Department of Histology and Embryology, Faculty of Veterinary Medicine, Istanbul University, Cerrahpaşa, 34320 Istanbul, Türkiye; abit@iuc.edu.tr; 6 Department of Histology and Embryology, Faculty of Medicine, Çukurova University, 01330 Adana, Türkiye; spolat72@gmail.com

**Keywords:** diabetes mellitus, *Garcinia kola*, curcumin, motor neuron, physical disector, neuroprotection

## Abstract

**Background/Objectives**: Diabetes mellitus is known to exacerbate neural degeneration and impair regenerative capacity following injury. This study aimed to investigate the effects of *Garcinia kola* and curcumin on spinal cord morphology and neural structure in a diabetic sciatic nerve injury model. **Methods**: Spinal cord tissues were obtained from male Wistar albino rats subjected to sciatic nerve transection and diabetes induction in a previously established experimental model. Animals had been assigned to five experimental groups (*n* = 7 per group): control group, transected sciatic nerve group, transected sciatic nerve + diabetes mellitus (DM), transected sciatic nerve + diabetes mellitus + curcumin (DM + Cur), and transected sciatic nerve + diabetes mellitus + *Garcinia kola* (DM + GK). Curcumin (300 mg/kg/day) and *Garcinia kola* (200 mg/kg/day) were administered orally. All animals were sacrificed at three months post-injury. Cervical (C3–C5) spinal cord segments were processed for histological evaluation, and quantitative assessments were performed using unbiased stereological methods to estimate neuronal and structural parameters. Microscopic analyses were conducted to assess morphological alterations. **Results**: Diabetes combined with sciatic nerve transection induced marked structural alterations in the spinal cord, including reductions in neuronal density and disruption of tissue organization. The DM group showed a significantly lower number of motor neurons than the other groups; similarly, it showed a significant decrease in soma area compared to the Cont and Sham groups. **Conclusions**: Both the curcumin and *Garcinia kola* treatment groups exhibited partial preservation of spinal cord morphology. This study provides evidence that *Garcinia kola* and curcumin exert protective effects not only at the peripheral nerve level, but also in the spinal cord following diabetic nerve injury.

## 1. Introduction

Diabetes mellitus (DM) is a metabolic disease characterized by hyperglycemia. Excessive food intake and a sedentary lifestyle contribute to the development of type 2 DM [1]. Diabetes also causes significant effects on the central nervous system [1,2]. It causes long-term cell death and functional impairment in the brain. Type 2 DM may increase the risk of cognitive dysfunction. Although the underlying mechanisms have not yet been fully elucidated, current findings indicate that vascular disorders and neurodegeneration that occur after diabetes lead to cognitive damage [3]. It is known that the brain is sensitive to oxidative damage caused by diabetes. A decrease in antioxidant enzyme levels and an increase in reactive oxygen and nitrogen species can lead to increased cell loss [4,5]. Moreover, increased malondialdehyde in brain tissue in a streptozotocin-induced diabetic rat model indicates that diabetes leads to lipid peroxidation in the central nervous system [6].

Although the effects of DM on the nervous system are mostly investigated at the brain level [4,5,7], most patients with DM develop a neurological picture that also affects the peripheral and cranial nerves and the spinal cord. Most of the time, the possibility of DM is considered in the differential diagnosis of patients who present to the clinic with neurological symptoms [8]. Diabetic neuropathy, one of the most common complications in individuals with DM, can lead to motor dysfunctions and physical disabilities as it affects the somatic and autonomic nerves [9,10]. In a diabetic rat model, diabetic neuropathy is characterized by decreased motor axon conduction velocity in the early phase [11,12]. In addition, susceptibility to diabetes-induced neurodegeneration was detected in the sensory part of the peripheral nervous system in animals with a long-term diabetes model [13]. Diabetic neuropathic pain, frequently seen in the peripheral nervous system, is equivalent to the severity of pain caused by spinal cord injury and demyelination [14]. Increased response to painful and painless stimuli is typical in patients with diabetes [15]. In diabetic patients, motor and cognitive systems become sensitive because of microvascular dysfunction, which may predispose them to neurological problems [16]. Even if motor neurons are resistant to the effects of diabetes, functional changes can still be observed [17]. Recently, it was suggested that diabetic peripheral neuropathy affects not only the brain but also the cervical spinal cord. Since systemic inflammation caused by diabetes leads to enlargement of the cervical spinal cord. Early inflammatory enlargement and late atrophy are observed in diabetic spinal cord pathology [18]. In this study, we aimed to examine the morphology of motor neurons in the cervical spinal cord at the cellular level using immunohistochemical and electron microscopic methods to clarify the effect of diabetes on motor neurons.

Various antioxidant polyphenols, such as curcumin, may be effective in stopping the progression of diabetic neuropathy [19]. In a previous study, 100 mg/kg/day of curcumin decreased TNF-alpha and interleukin-10 levels after 6 weeks of treatment [20]. As a result of the administration of 200 mg/kg/day curcumin in STZ-induced rats for 14 days, decreased hydrogen peroxide levels were observed in the spinal cord [21]. There is evidence for curcumin’s neuroprotective effect on the central nervous system [22,23]. It was observed that the neurons in the lumbar region of the spinal cord decreased, and lipid peroxidation occurred in transgenic mice that developed an Alzheimer’s disease model. Reversal of motor function deficits was observed in these mice due to curcumin treatment [24]. However, no data were available on the curative effect of curcumin on cervical motor neurons in diabetic rats. Another plant that has attracted attention in recent years with its neuroprotective properties is *Garcinia kola*. In the STZ-induced diabetic mouse model, the effect of *Garcinia kola* on fine motor skills was examined. It was observed to contribute to the development of motor symptoms [25]. Although the effect of diabetes on motor functions has been partially addressed in the literature [25,26], morphological research on its possible protective effect on cervical motor neurons after diabetes is limited. In this study, we focused on two main hypotheses. First, we aimed to investigate possible degenerative effects using immunohistochemical and light microscopic methods, assuming that motor neurons are susceptible to neurodegeneration in diabetic rats with transected sciatic nerve injury. Our second assumption is that *Garcinia kola* and curcumin may show neuroprotective activity against possible motor neuron degeneration. Curcumin has a very low capacity to cross the blood–brain barrier. Its oral bioavailability is low, and its metabolism is rapid [27]. Mechanistic research into the role of *Garcinia kola*’s ability to cross the blood–brain barrier in its neuroprotective activity is insufficient [28]. Therefore, both antioxidants may exert neuroprotective effects through their anti-inflammatory activity. It can be suggested that *Garcinia kola* and curcumin may be effective through both systemic and central mechanisms. Besides this explanation, this issue remains to be solved.

In summary, exploratory investigations of the neuroinflammatory effects of sciatic nerve injury suggest that it also affects the cervical segments of the spinal cord [29]. The effects of systemic inflammation, such as diabetes mellitus, on sciatic nerve damage can be examined; the effects of this metabolic disease on the C_3_–C_5_ cervical spinal cord can be evaluated. Such inflammation may lead to astrocyte activation even in distant spinal cord segments. In the presence of diabetes, a more widespread neuroinflammatory effect may be observed not only in the lumbosacral region but also in the cervical spinal cord. The present study aimed to address the possible distant neuroinflammatory changes resulting from sciatic nerve injury and, conversely, to investigate the therapeutic effects of *Garcinia kola* and curcumin.

## 2. Results

### 2.1. Soma Area and Motor Neuron Number Results

**Figure 1:**
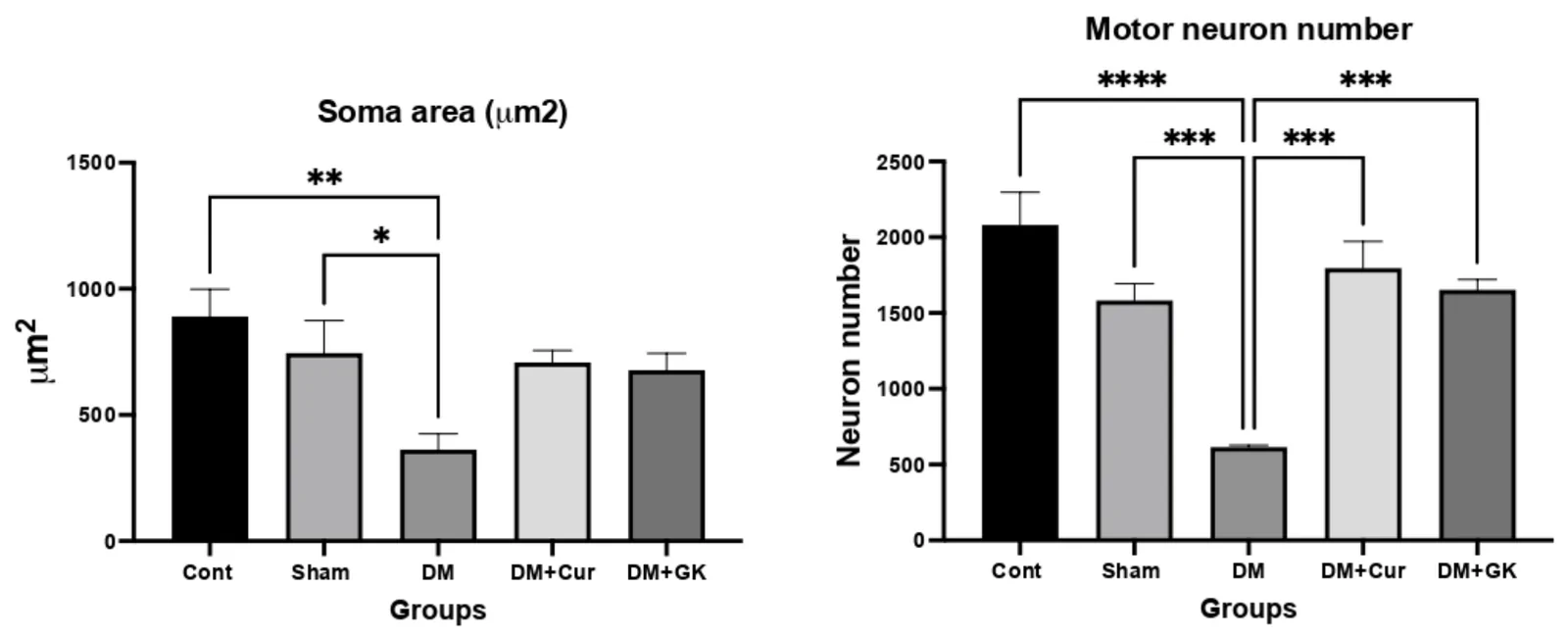
Quantitative analysis of spinal cord motor neuron number and soma size across experimental groups. (**Left**) Soma cross-sectional area (µm^2^) of spinal cord motor neurons in Cont, Sham, DM, DM + Cur, and DM + GK groups. Total number of motor neurons in the spinal cord per group. Data are presented as mean ± SD. A decrease in soma area values was detected in the DM group compared to the Sham and Cont groups (*p* < 0.005 and *p* < 0.001, respectively). (**Right**) Similarly, a decrease in motor neuron number was found compared to the Sham and Cont groups (*p* < 0.0001 and *p* < 0.00001, respectively). In addition, a decrease was detected in the DM group compared to the DM + Cur and DM + GK groups (*p* < 0.0001). Asterisks indicate significant differences between groups (* *p* < 0.05, ** *p* < 0.01, *** *p* < 0.001, and **** *p* < 0.0001). Statistical analysis was performed using one-way ANOVA (post hoc Tukey’s test) and the Kruskal–Wallis test (post hoc: Dunn’s test).

No statistically significant difference was observed between the Cont and Sham groups (*p* = 0.1047). In the DM group, the mean motor neuron soma area was approximately 59.3% lower than in the control group (*p* < 0.0001) and approximately 51.4% lower than in the Sham group (*p* = 0.0008). These reduction rates confirm significant neuronal atrophy associated with diabetes. No significant differences were detected between Cont and DM + Cur (*p* = 0.6098) or Cont and DM + GK (*p* = 0.2403), suggesting that both curcumin and *Garcinia kola* treatments restored soma area toward normal physiological levels. Similarly, comparisons between the sham and both treatment groups were not statistically significant, further supporting treatment-induced normalization of neuronal morphology. Considering the motor neuron number, the control group differed significantly from the DM group (*p* < 0.0001), indicating a strong diabetes-related effect. No significant differences were observed between the Cont and DM + Cur (*p* = 0.6098) or the Cont and DM + GK (*p* = 0.2403) groups, suggesting that both treatments restored the measured parameter toward control levels. Similarly, no significant differences were detected between the Sham group and treatment groups (*p* = 0.9952). In contrast, the Sham group differed significantly from the DM group (*p* = 0.0008), further confirming the detrimental effect of diabetes. Direct comparisons between diabetic and treated groups showed statistically significant differences for both interventions (DM vs. DM + Cur: *p* = 0.0002; DM vs. DM + GK: *p* = 0.0006), indicating that both treatments significantly improved outcomes compared to untreated diabetes. No statistically significant difference was found between the DM + Cur and DM + GK groups (*p* = 0.9476), indicating comparable effects of the two therapeutic interventions (Figure 1).

Table 1 summarizes the estimated total number of spinal motor neurons and mean soma cross-sectional area obtained from the Cont, Sham, Diabetes Mellitus (DM), DM + Curcumin (DM + Cur), and DM + *Garcinia kola* (DM + GK) groups. Values are presented as mean ± SD. Precision of stereological estimates is indicated by the coefficient of error (CE), and biological variability within each group is represented by the coefficient of variation (CV).

### 2.2. Mean Cross-Sectional Area and WM/Total and GM/Total Ratio Results

**Figure 2:**
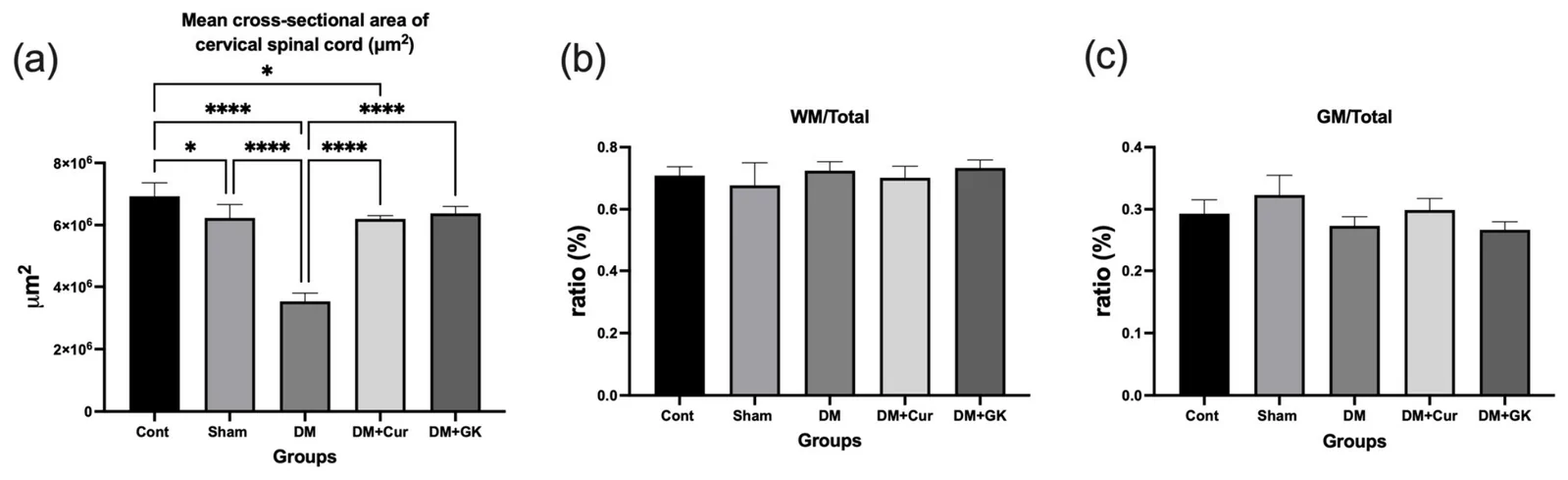
(**a**) Post hoc analysis using Tukey’s multiple comparisons test showed that the DM group differed significantly from both the Cont and Sham groups (*p* < 0.0001 for both), demonstrating a pronounced diabetes-related reduction in the mean cross-sectional area. No significant differences were found between the Cont and treatment groups (Cont vs. DM + Cur: *p* = 0.0808; Cont vs. DM + GK: *p* = 0.2780), nor between the Sham group and treatment groups (Sham vs. DM + Cur: *p* = 0.9885; Sham vs. DM + GK: *p* = 0.9981), indicating normalization of mean cross-sectional area following treatment. Direct comparisons confirmed significant differences between the untreated diabetic group and both treatment groups (DM vs. DM + Cur: *p* < 0.0001; DM vs. DM + GK: *p* < 0.0001), while no significant difference was observed between the two treatment groups (DM + Cur vs. DM + GK: *p* = 0.9452), indicating comparable efficacy. (**b**,**c**) Analysis of the GM/Total and WM/Total ratios revealed no statistically significant differences between the experimental groups. * *p* < 0.05, and **** *p* < 0.0001. Statistical analysis was performed using one-way ANOVA (post hoc Tukey’s test) and the Kruskal–Wallis test (post hoc: Dunn’s test).

When the groups were examined in terms of the mean cross-sectional area of the cervical spinal cord, the mean cross-sectional area in the DM group was significantly reduced compared to both the control and Sham groups (*p* < 0.0001) (Figure 2a). Furthermore, the DM group had significantly lower values compared to the DM + Cur and DM + GK groups (*p* < 0.0001). However, no statistically significant difference was observed between the control group and the DM + Cur and DM + GK groups, nor between the Sham group and the treatment groups. No significant difference was found between the DM + Cur and DM + GK groups either (*p* > 0.05). When the white matter/total tissue (WM/Total) and gray matter/total tissue (GM/Total) ratios were evaluated, no statistically significant difference was observed between the experimental groups (*p* > 0.05) (Figure 2b,c).

Table 2 summarizes stereological estimations of mean total spinal cord section area, white matter to total tissue ratio (WM/Total), and gray matter to total tissue ratio (GM/Total) in Cont, Sham, Diabetes Mellitus (DM), DM + Curcumin (DM + Cur), and DM + *Garcinia kola* (DM + GK) groups. The reliability and precision of stereological estimations are indicated by the coefficient of error (CE), while biological variability among animals within each group is represented by the coefficient of variation (CV).

### 2.3. Histopathological Evaluation

**Figure 3:**
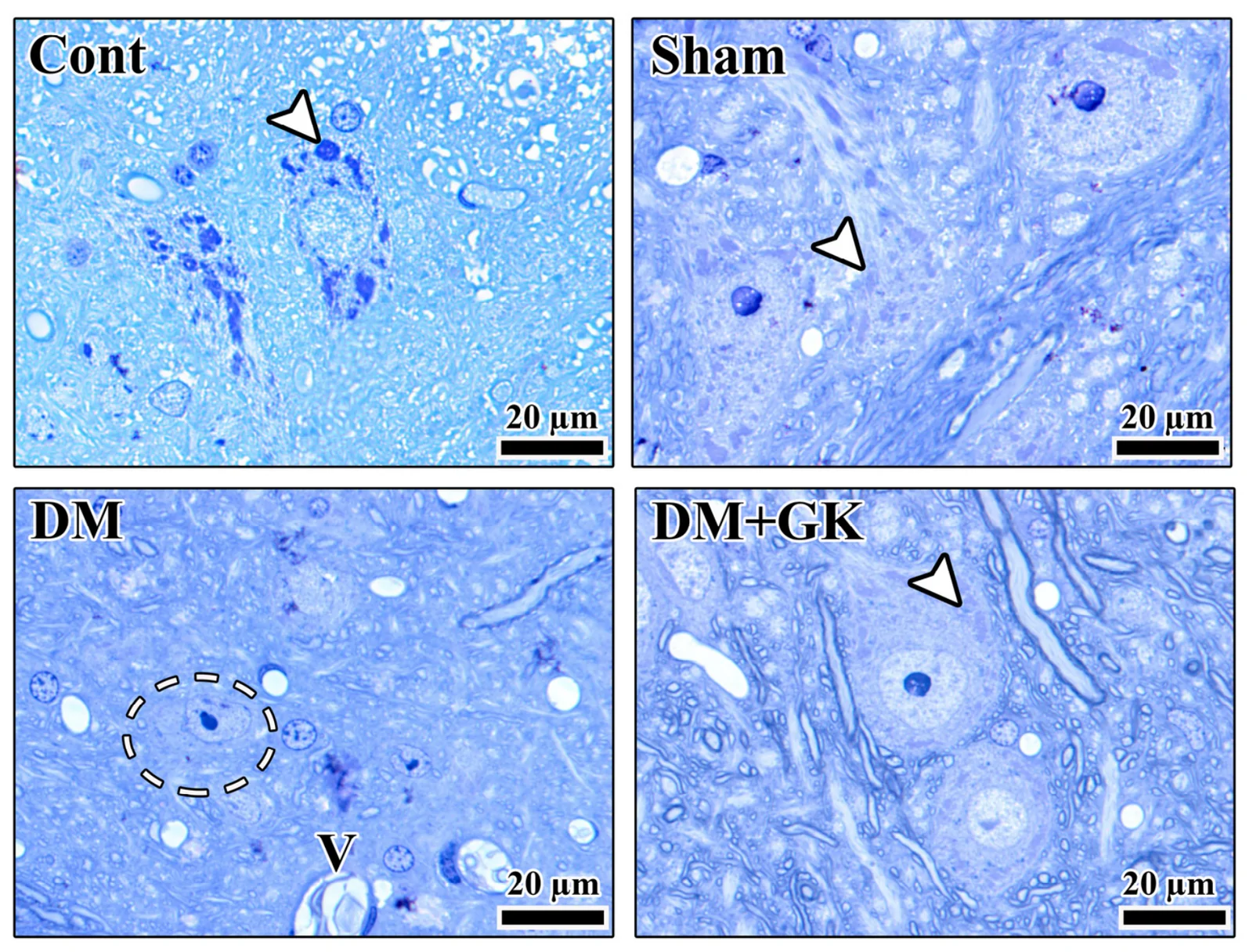
The images obtained from semithin sections of the groups are shown. Arrowheads indicate Nissl bodies in the cell bodies of motor neurons. In the DM group, the soma structure of the contracting motor neuron is shown within a striated circle. Motor neurons in the DM + GK have a preserved morphology. Resin section, toluidine blue staining. Scale bars: 20 µm.

Examination of toluidine blue-stained sections from resin blocks revealed that in the Cont group, the nerve tissue had a normal morphology, with distinct neuronal cell bodies and regularly arranged fibers surrounding them. Nissl bodies, which stained darker compared to other groups, were observed in the Cont group. Similarly, in the Sham group, the histological structure was largely preserved, and no significant pathological findings were observed. In the DM group, no extensive neuronal degeneration was observed; however, neuronal cell bodies lacked boundaries, and Nissl bodies were not visible. On the other hand, deterioration of the walls of the vascular structures in the tissue was observed. In the DM + GK group, a healthier histological structure was observed compared to the DM group. Neuronal structures were found to be more regularly arranged in the DM + GK group (Figure 3).

### 2.4. Electron Microscopic Assessment

**Figure 4:**
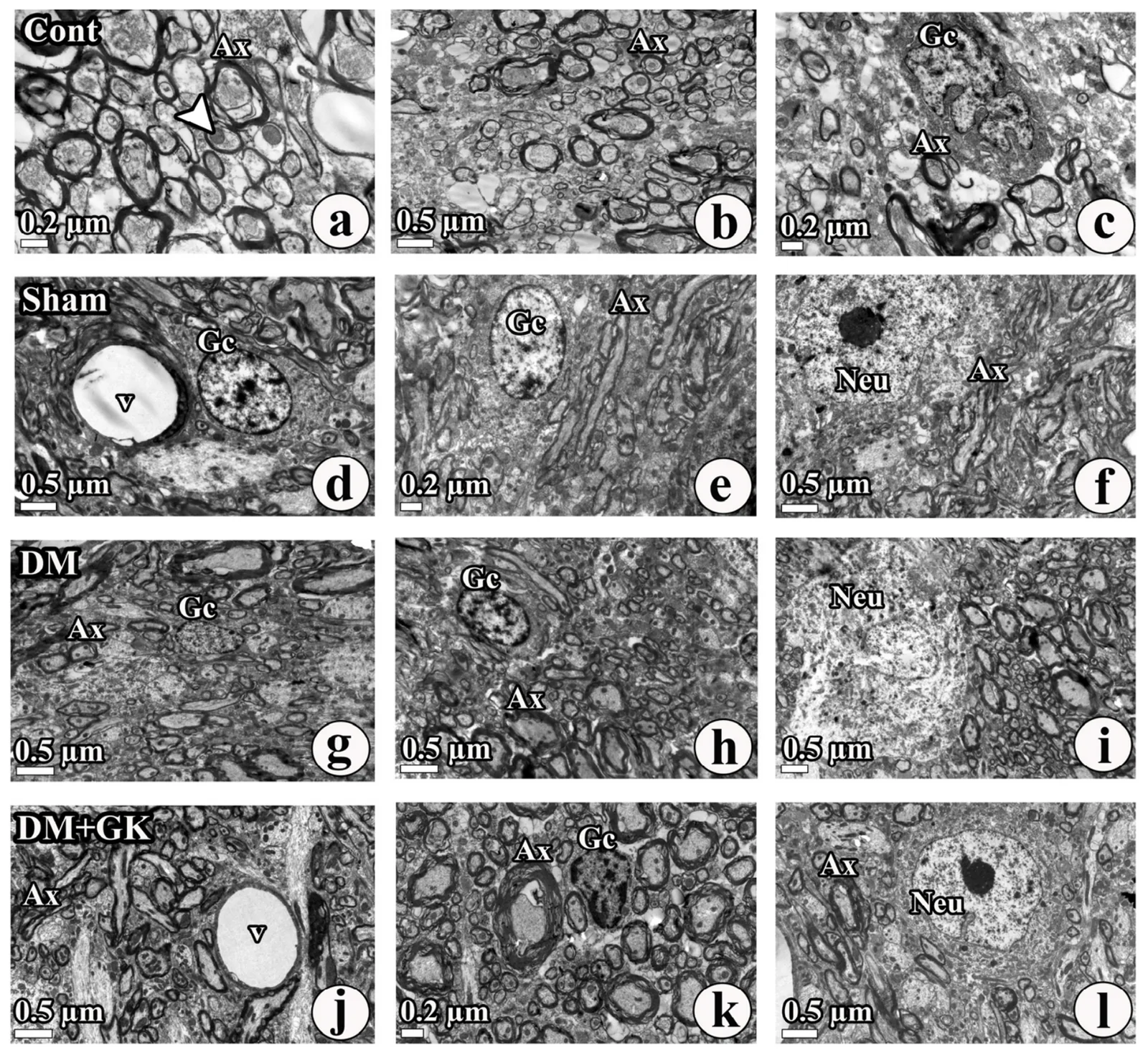
Electron microscopic images of the groups are shown. (**a**–**c**) Images of the Cont group show a preserved ultrastructure. Mitochondria (arrowheads) in the axon cytoplasm are observed in normal structure. (**c**) The cell with a heterochromatic nucleus is likely microglia. (**d**–**f**) In images of the Sham group, cellular structures and myelin sheaths of myelinated axons appear preserved. (**g**–**i**) Images of the DM group show that the myelin sheath is thinner than in the Cont group. Borders of structures are not delineated. (**i**) Nucleus and nucleolar boundaries are not clearly observed in the neuronal structure. This may indicate that the neuron is undergoing degeneration. (**j**–**l**) Images from the DM + GK group show axons, glial cells, and neurons. Most of the structures are seen in preserved morphology. Resin section, uranyl acetate and lead citrate staining. Scale bars: 0.2 µm, 0.5 µm.

Electron microscopic images revealed preserved morphological features in the neuronal, glial, and nerve fibers in the Cont group; mitochondria in the axon cytoplasm were normal. The heterochromatic structure of glial cells was noteworthy in this group. In the Sham group, electron microscopy revealed vacuolization in some areas of the myelin sheath of myelinated axons. However, no significant structural damage was observed in glial cells and neurons. In the DM group, myelin sheath damage and vacuolization were noticeable. Furthermore, the boundaries between the nucleus and nucleolus were not clearly visible in the neuron. Neuronal structures undergoing apoptotic processes were observed in this group. In the DM + GK group, the axon, glial cell, and neuron structures were generally better preserved compared to the DM group. However, vacuolization was observed in the myelin sheaths of some axons. The presence of axonal shrinkage was also noted in some axons. This indicated a partial recovery from axonal degeneration (Figure 4).

### 2.5. Immunohistochemical Evaluation

**Figure 5:**
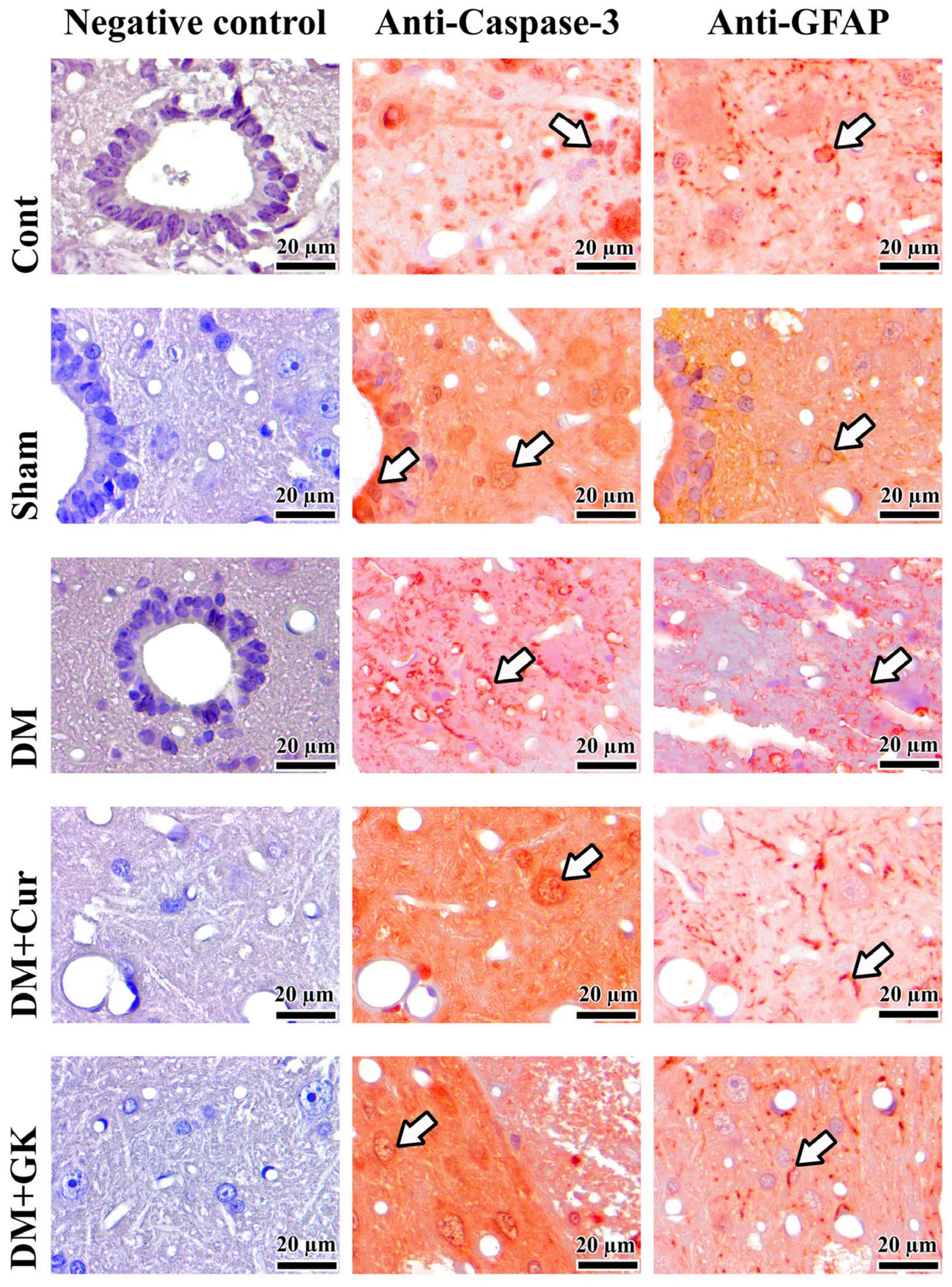
Immunohistochemical staining with anti-caspase-3 and anti-GFAP antibodies is shown for all groups. Cells showing a positive reaction are indicated by arrows. No contamination was found in the negative controls. Paraffin sections: Mayer’s hematoxylin was used for reverse staining. Scale bars: 20 µm.

When the groups were examined for anti-GFAP and anti-caspase-3 immunoreactivity, no contamination was observed in the negative control sections. When the anti-GFAP-stained sections were examined, physiological-level astrocytic staining was observed in the Cont group. In the Sham group, more intense positive staining was observed compared to the Cont group. Anti-GFAP staining was less intense in the DM group than in the Cont and Sham groups. In the sections belonging to the DM + Cur group, normal levels of anti-GFAP antibody expression were observed. Reactive astrocytes were observed in this group, but at a lower level than in the DM + GK group. The anti-GFAP intensity in the DM + GK group was similar to the reactivity in the Cont group. When sections from the groups were evaluated for anti-caspase-3 expression, a physiological level was observed in the Cont group, whereas intense expression was observed in the Sham, DM + GK, and DM + Cur groups. In the DM group, however, anti-caspase-3 expression was more pronounced in the axons than in the nuclei (Figure 5).

## 3. Discussion

DM causes neuronal damage and impairs regenerative capacity [30]. Diabetic neuropathy leads to motor dysfunctions [31], and effects on the dorsal and ventral horns of the spinal cord can result in distinct neurological outcomes. The cellular effects of hyperglycemia in the dorsal horn can lead to degeneration of the central nervous system. On the other hand, studies examining the effects of diabetes on the ventral horn are limited [30]. In a study on motor neurons, it is noteworthy that, although there are structural changes at the cellular level due to diabetes, there is no numerical decrease. Data on the effects of diabetes on spinal motor neurons are not clear in the literature. Ramji et al. showed that although stress-related parameters increased in the cytoplasm of motor neurons, the number of motor neurons did not decrease [12]. On the other hand, studies have suggested that not only the number of motor neurons but also their diameters decreased in diabetes [10,32]. In our study, the effects of diabetic polyneuropathy were discussed at both the peripheral nervous system and the cervical spinal cord level. As stated, the effects of hyperglycemia on the number and volume of spinal motor neurons have been discussed. In the present study, we investigated the effects of long-term diabetes on motor neurons in the cervical spinal cord following combined long-term diabetes and sciatic nerve transection.

Caspase-3 plays an important role in both physiological and pathological apoptosis [33]. Immunohistochemical analysis of anti-caspase-3 activity was performed in the DM group. In this regard, findings of anti-caspase-3 immunoreactivity indicate significant differences in apoptosis levels between the experimental groups. The physiological level of caspase-3 expression observed in the Cont group is consistent with normal cellular cycling and basal apoptotic activity. In contrast, the increased caspase-3 immunoreactivity in the Sham group may reflect activation of apoptotic pathways induced by surgical stress. The high caspase-3 positivity observed in the treatment groups (DM + Cur and DM + GK) suggests that these agents may have limited effects on apoptosis mechanisms in a diabetic setting. High levels of Caspase-3 immunoreactivity were observed in the treatment group. This finding should not be interpreted as indicating a lack of neuroprotective effect. Previous studies have shown that transient or sublethal Caspase-3 activation may be part of adaptive cellular responses as well as apoptosis [34]. Therefore, the persistent Caspase-3 immunoreactivity observed in the treatment group in our study may be indicative of an adaptive cellular process rather than irreversible neuronal degeneration. However, the underlying mechanisms need to be elucidated. The changes in caspase-3 expression observed in the DM group may have been triggered by oxidative stress associated with hyperglycemia. These findings demonstrate that diabetes increases the risk of cellular loss in the central nervous system and that apoptotic processes are modulated to varying degrees between experimental groups. Apoptotic processes were evaluated using curcumin and *Garcinia kola* treatment. In the present study, we observed that oxidative stress, apoptosis, and inflammation processes have a role in the pathological changes in the cervical spinal cord caused by diabetes. As previous experimental studies have shown, the present study suggests that the pathological changes in the cervical spinal cord caused by diabetes may be related to apoptosis and inflammation.

In an STZ-induced diabetic rat model, decreased GFAP levels were detected in enteric glial cells of the digestive system [35]. In our study, we observed a decrease in GFAP expression in the DM compared to the Cont group. In this regard, our finding is consistent with this study. On the other hand, GFAP levels have been considered as a marker associated with the progression of type 1 and type 2 diabetes in animal models of diabetes [36]. In this context, it is suggested that the effects of agents used to treat diabetes on GFAP expression are important and that this idea could lead to new therapeutic strategies. The anti-inflammatory effects of curcumin may be achieved by modulating astrocyte function [37]. A previous study suggested that cognitive and motor impairments associated with type 1 DM are largely due to neuroinflammation and glial activation. Related to this, *Garcinia kola* has been reported to normalize the glial response by suppressing GFAP increase [26]. In our study, *Garcinia kola* did not suppress GFAP; in fact, GFAP expression increased. In the DM + GK group, the higher GFAP immunoreactivity intensity compared to the control group may be dose- and duration-dependent. While the increase in GFAP in the GK-treated group cannot be interpreted solely as astrocyte activation, this increase may also indicate different biological processes [38,39]. In this context, in our study, this increase in the GK group may also be related to reactive astrogliosis, which is associated with supporting neuronal survival.

The physiologically high GFAP expression observed in the Cont group represents normal astrocyte integrity and basal glial activity. In contrast, the increased GFAP staining intensity in the Sham group may suggest that surgical stress on the sciatic nerve may trigger astrocyte activation in the spinal cord. This finding may indicate that astrocytes are sensitive to environmental stimuli and that GFAP expression may increase in response. A decrease in GFAP immunoreactivity in the DM group compared to the Cont and Sham groups may indicate that diabetes may be associated with both reactive astrogliosis and loss of astrocyte function or structural damage [40]. It can be suggested that oxidative stress and inflammatory processes due to hyperglycemia in DM suppress GFAP synthesis in astrocytes. This suggests that in DM, the glial response may be suppressed over time, and neuronal support functions may decrease. When expression levels in the treatment groups were evaluated, GFAP expression in the DM + Cur group approached physiological levels and was similar to that in the Cont group, suggesting that curcumin partially corrects diabetes-related astrocytic damage and stabilizes the glial response.

When the stereological data from the present study were evaluated, it was observed that experimental diabetes caused significant neurodegenerative effects on motor neurons in the cervical spinal cord, resulting in significant reductions in motor neuron number, somatic cross-sectional area, and mean cross-sectional area of the spinal cord. On the other hand, curcumin and *Garcinia kola* applications largely prevented these diabetes-related structural damages, preserving motor neuron morphology and spinal cord integrity. One of the most striking quantitative findings of the study is the significant decrease in motor neuron number observed in the diabetic group. Motor neuron loss points to the destructive effect of diabetes at the cervical level of the spinal cord. In addition to the decrease in motor neuron number, a significant reduction in somatic cross-sectional area was found in the DM group. The decrease in somatic area may stem from cytoskeletal destruction occurring in neurons under metabolic stress [41]. In this regard, further molecular-level studies are needed. When stereological and histopathological findings are evaluated together, it can be suggested that diabetes causes not only neuronal loss but also morphological degeneration in motor neurons and throughout the spinal cord. When stereological data from DM + Cur and DM + GK groups were evaluated, curcumin administration showed that both the number of motor neurons and the soma area approached control levels, revealing the herb’s neuroprotective properties. The findings of this study demonstrate the structural protection of curcumin on spinal cord motor neurons. Similarly, *Garcinia kola* administration significantly reduced diabetic-related motor neuron loss and soma deformities.

When volumetric data were examined, the diabetic group showed a significant decrease in the mean cross-sectional area of the cervical spinal cord, which may be due to widespread neurodegeneration associated with diabetes. The fact that the mean cross-sectional area of the spinal cord in the curcumin and *Garcinia kola* groups was similar to that in the Cont group may show that these therapeutic agents contributed to improvements in histomorphological structure. On the other hand, no significant difference was observed between the groups in the ratios of gray matter and white matter to the total cross-sectional area of the spinal cord. Diabetes may affect the gray and white matter of the cervical spinal cord in a proportional manner.

The evaluation of the cervical spinal cord region only and the absence of evaluations in the lumbosacral spinal cord can be considered limitations of the study. In this study, given the limited information in the current literature on the effects of peripheral nerve damage on the cervical spinal cord, the investigation of neuroinflammatory effects in this region was undertaken. Among the limitations of our study, it can be said that only the effects on male rats were examined; no studies were conducted on female rats. Therefore, these findings cannot be generalized to female animals. On the other hand, sex-specific differences need to be discussed in future studies. Also, stereological findings were not supported by molecular analyses. In summary, this study reveals that diabetes causes significant impairment of spinal motor neurons at both the numerical and morphological levels, while curcumin and *Garcinia kola* exhibit neuroprotective effects, largely preventing this damage. Since curcumin and *Garcinia kola* extract have different pharmacological profiles, bioavailabilities, and mechanisms of action, the doses reported in the literature as most accurate for their neuroprotective efficacy were selected. Furthermore, different application durations and doses were designed to ensure both compounds are effective. In this context, the study evaluates the significant role of phytotherapeutic agents in preventing or improving diabetic motor neuropathy. Further studies using advanced molecular analyses are needed to evaluate the effects of diabetes on motor neurons and to further elucidate the neuroprotective effects of these agents. Immunohistochemical evaluations reflecting the total amount of caspase-3 may also represent a physiological process. Another limitation of this study is the inability to obtain sufficiently high-quality sections from the resin blocks of the DM + Cur group for histopathological evaluation.

## 4. Materials and Methods

### 4.1. Animals, Ethical Considerations, and Experimental Groups

Experimental procedures in our study were performed with the approval of the Ondokuz Mayıs University Animal Experiments Local Ethics Committee (approval number: 2019/49). All surgical and experimental procedures were performed in accordance with ARRIVE and the Guide for the Care and Use of Laboratory Animals.

In this study, adult male Wistar albino rats weighing 250–300 g were used. Rats were randomly divided into five groups (*n* = 7). For the present study, no separate sample size (power) analysis was performed a priori. The statistical power of the study was determined using G*Power software (version 3.1.9.7; Heinrich Heine University, Düsseldorf, Germany). Power analysis was performed based on neuron soma area; α = 0.05, Cohen’s f = 0.963, power = 0.9946. Rats in stainless steel cages were subjected to a 12 h dark/12 h light cycle. Rats were housed at optimal room temperature (22 ± 2 °C) and optimal humidity (50–60%). The rats had free access to water and pellet food. A detailed definition of all groups is given in Table 3.

### 4.2. Diabetes Induction

In this study, adult rats in the DM, DM + GK, and DM + Cur groups were injected with a single dose of streptozotocin (50 mg/kg; Sigma-Aldrich, S0130, St. Louis, MO, USA) dissolved in citrate buffer (0.1 M, pH 4.5). During the experiment, rats injected with STZ in the morning were fasted overnight; those injected in the afternoon were fasted for at least 4 h before injection. During the injection, the syringe tip was checked to ensure that the needle did not enter the intestine, bladder, or a vessel [42]. Glucose levels in blood samples collected 72 h after STZ injection were measured using Accu-Chek glucometers (Roche Diagnostics GmbH, Mannheim, Germany). Rats with a blood glucose level above 250 mg/dL were considered “diabetic.” Weekly body weights and blood glucose values of the rats were followed [43].

### 4.3. Garcinia kola Analyses

Dried *Garcinia kola* seeds from Nigeria were purchased in Makkah (Saudi Arabia). The seeds were authenticated by botanists of Qassim University College of Agriculture, and samples were stored at the Department of Histology and Embryology, Ondokuz Mayıs University. Seed suspension was prepared daily and used immediately [44].

The analysis was performed using ultrasound-assisted extraction with 70% ethyl alcohol. For this purpose, 2.5 g of the sample was taken, 50 mL of 70% ethyl alcohol was added, and the mixture was first placed in an ultrasonic water bath at 20 °C for 30 min, then in the refrigerator at 4 °C for 12 h. To remove turbidity, the mixture was centrifuged at 3000 rpm for 15 min. Before analysis, the samples were passed through a 0.45 µm Teflon filter.

### 4.4. Total Phenolic Content

The method reported by [45] was modified and used to determine the total phenolic content. In total, 0.5 mL of the sample extracted with ethyl alcohol and diluted to the appropriate ratio was mixed with 2.5 mL of Folin–Ciocalteu reagent and left to stand for 5 min [43]. Then, a 7.5% sodium carbonate solution was added, and the sample was left in a dark environment for 2 h. At the end of this period, it was read against a standard curve of pure gallic acid at 760 nm, and the total phenolic content was calculated (Table 4).

### 4.5. Iron Reducing Antioxidant Potency (FRAP)

Samples extracted with ethyl alcohol were diluted to the appropriate ratio and mixed with a 10:1:1 (*v*:*v*:*v*) mixture of 300 mM acetate buffer: 20 mM FeCl3: 10 mM 2,4,6-tripyridyl-s-triazine (TPTZ) solution (dissolved in 40 mM HCl). The absorbance was determined at 593 nm using a spectrophotometer. A calibration curve was plotted, and the results were calculated using solutions prepared with Trolox [45].

### 4.6. Determination of the Scavenging Effect of DPPH Free Radical

The method reported by Tural and Koca (2008) was modified and used to determine the scavenging effect of the DPPH radical (2,2-diphenyl-1-picrylhydrazyl) [46]. Fifty µL of the extract prepared with ethyl alcohol was mixed with 1 mL of 100 µM DPPH solution and kept in the dark at room temperature until the reaction was completed, and its absorbance at 517 nm was determined. In addition, the same procedures were performed by preparing a control sample with ethanol, and the DPPH radical scavenging effect was calculated using the formula below. A calibration curve was plotted, and the results were calculated using solutions prepared with Trolox.

DPPH inhibition (%) = ((Absorbance of control sample − Absorbance of sample)/Absorbance of control sample) × 100

### 4.7. Surgical Procedure and Vein Graft

All surgical procedures performed on the rats were conducted under anesthesia. During the procedures, the rats’ respiratory rates remained stable. For anesthesia, rats were administered 50 mg/kg ketamine (Ketalar, Eczacıbaşı, İstanbul, Turkey) and 20 mg/kg xylazine (Rompun, Bayer, İstanbul, Turkey) intraperitoneally. During the transection of the sciatic nerve, the 10 mm long nerve tissue 2 cm distal from the sciatic notch was removed. The jugular vein applied to the transected nerves was obtained by cutting the midline of the neck. Without puncturing the pectoralis muscle, the posterior and anterior facial veins were excised. The proximal and distal epineural sheaths of the sciatic nerve were anastomosed as a vein graft (11.3 ± 2.1 mm) [47]. Following the surgical operation, rats were injected with cefazolin sodium for three days to prevent possible infections. The rats were kept in separate cages during the recovery period.

### 4.8. Perfusion, Tissue Processing, Sectioning, and Stereological Analyses

The cervical spinal cord (C3–C5) and sciatic nerve tissues obtained after cardiac perfusion were fixed in a 4% glutaraldehyde + 4% paraformaldehyde mixture and embedded in resin after electron microscopic tissue follow-up. In addition, cervical spinal cord samples from each group were embedded in paraffin blocks for immunohistochemical analysis. The 0.5 µm-thick sections from the resin-embedded blocks were stained with toluidine blue, and light microscopy was performed. Electron microscopic examinations were used to detect cellular-level changes in motor neuron structure. Quantification of motor neurons in the ventral horn was carried out using the unbiased physical disector method using a stereological approach. Image acquisition and analysis were performed using a Nikon Eclipse 600 light microscope (Nikon Corporation, Tokyo, Japan) and ImageJ (version 1.54, National Institutes of Health, Bethesda, MD, USA). Stereological analyses were designed to examine neuron number and gray matter to white matter volume ratios. Morphoquantitative analyses aimed to investigate the effects of surgical procedures, diabetes, and applied treatments on neuron number.

**Figure 6:**
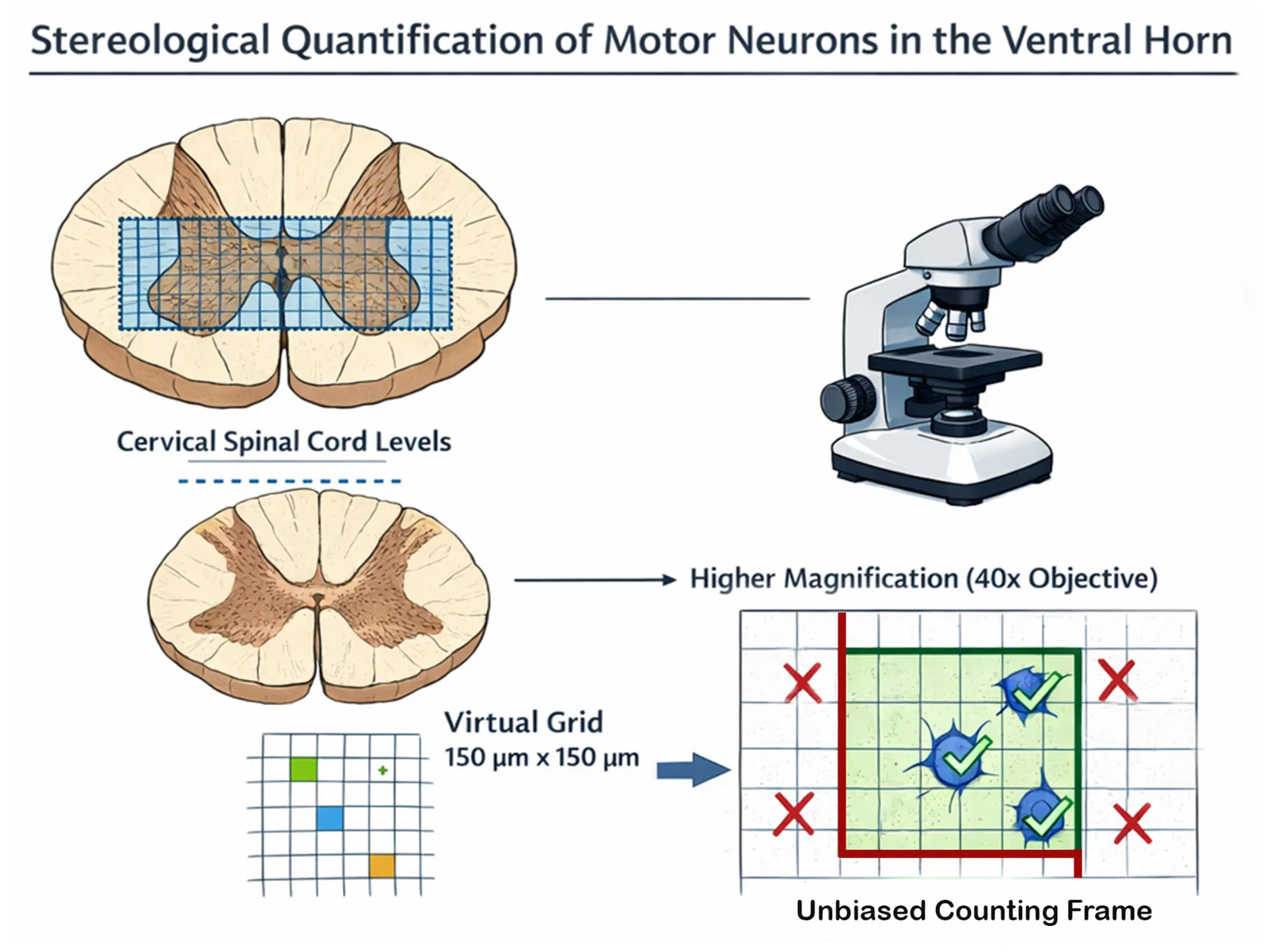
Schematic illustration of the unbiased physical disector approach used to estimate motor neuron numbers in the ventral horn of the cervical spinal cord. This figure was generated using ChatGPT (OpenAI, GPT-5.6 Sol) AI-based illustration tool for schematic purposes only. According to the main rule of the unbiased counting framework, a green check mark indicates nuclei included in the count, while a red X symbol indicates nuclei excluded from the count.

Systematic random sampling was implemented using a virtual grid spacing of 150 μm × 150 μm and an unbiased counting frame measuring 75 μm × 75 μm. These parameters were optimized to achieve a minimum of 200 counted neurons per animal, resulting in coefficients of error below 0.05. Spinal cord regions were first outlined at low magnification using a 10×/0.45 objective, followed by motor neuron counting at higher magnification with a 40×/1.40 NA objective. Neurons were identified within lamina IX based on morphological criteria, including a soma diameter between 20 and 25 μm, the presence of a distinct nucleus, and an identifiable mean orthogonal diameter. Sampling was performed across cervical levels C3–C5, with 15 sections analyzed per level and spinal cord segment, at approximately 120 μm intervals. Data were reported as mean motor neuron counts per ventral horn, including both hemisides of the spinal cord (Figure 6). Beyond numerical estimation, motor neuron morphology within the cervical anterior horn was also evaluated. Morphometric analyses were conducted using the physical disector methodology and unbiased counting frame implemented in ImageJ, in accordance with the fundamental principles of unbiased stereology.

### 4.9. Immunohistochemistry

Immunohistochemical analyses were performed to assess astrocyte activation and apoptosis. For the analysis of the expression of anti-glial fibrillary acidic protein (GFAP) (Abcam, Cambridge, UK) and anti-caspase 3 (Abcam, Cambridge, UK) antibodies in the cervical spinal cord, 4 µm thick sections were taken from paraffin-embedded blocks. After deparaffinization, immunohistochemical staining was performed using the mouse-specific HRP/AEC (ABC) IHC Detection Kit (AB93705, Abcam, Cambridge, UK). Mayer’s hematoxylin was used for reverse staining. Five sections per group were used for immunohistochemical evaluation. Immunohistochemical scoring was performed on the samples from the groups, and the results for each group were expressed as “mean ± SD.”

### 4.10. Statistics

Statistical analyses were performed with GraphPad Prism version 8.00 (GraphPad Software, La Jolla, CA, USA), and a *p* value of < 0.05 was considered statistically significant. One-way ANOVA (Tukey’s post hoc test) was used for normally distributed data, and the Kruskal–Wallis test was used for data that did not show normal distribution.

## 5. Conclusions

It was observed that sciatic nerve transection in a diabetic rat model resulted in various morphological changes in the cervical spinal cord. It is believed that this may lead to multiple pathological outcomes, particularly those related to glial activity and glial cell function. Furthermore, the cervical spinal cord remodulation resulting from sciatic nerve transection may have prevented the effects of diabetes from manifesting. Curcumin was more effective at regulating glial activity, whereas *Garcinia kola* had limited effects. Furthermore, increased caspase-3 expression in the DM + Cur group suggests the induction of adaptive stress mechanisms in the cervical spinal cord. To further elaborate on the stereological and immunohistochemical results obtained in this study, a cellular investigation of apoptotic pathways in the cervical spinal cord after DM is necessary.

## Figures and Tables

**Table 1:** Stereological estimates of spinal motor neuron number and soma area in experimental groups. CE, coefficient of error; CV, coefficient of variation.

	Cont	Sham	DM	DM + Cur	DM + GK
Estimated motor neuron number	2081 ± 431	1582 ± 249	615 ± 26	1796 ± 355	1653 ± 137
CE	0.05	0.06	0.08	0.08	0.07
CV	0.20	0.16	0.13	0.20	0.08
Soma area (µm^2^)	890 ± 215	745 ± 256	362 ± 153	707 ± 95	676 ± 133
CE	0.05	0.06	0.07	0.06	0.4
CV	0.24	0.34	0.42	0.13	0.20

**Table 2:** Stereological estimates of total spinal cord volume and gray/white matter ratios in experimental groups.

	Control	Sham	DM	DM + Cur	DM + GK
Mean cross-sectional area (mm^2^)	0.0068 ± 0.0	0.0063 ± 0.03	0.0038 ± 0.0	0.0061 ± 0.0	0.0063 ± 0.02
CE	0.06	0.05	0.06	0.05	0.06
CV	0.2	0.6	0.16	0.18	0.30
WM/Total (%)	0.70 ± 0.02	0.67 ± 0.07	0.72 ± 0.03	0.70 ± 0.03	0.73 ± 0.02
CE	0.06	0.1	0.07	0.07	0.04
CV	0.04	0.11	0.04	0.05	0.04
GM/Total (%)	0.29 ± 0.04	0.32 ± 0.07	0.27 ± 0.02	0.29 ± 0.03	0.26 ± 0.02
CE	0.06	0.05	0.2	0.4	0.8
CV	0.15	0.22	0.11	0.12	0.10

**Table 3:** All study groups (*n* = 7).

Control Group	No Surgical Procedure or Treatment in This Group
Sham group	In this group, a 10 mm nerve was removed 2 cm distal to the sciatic nerve, and a vein graft was applied. During the 90-day recovery period, the rats were kept in separate cages.
Transected + Diabetes mellitus group (DM)	In this group, a diabetic model was created by injecting a single dose (STZ) (Sigma Aldrich, S0130, St. Louis, MO, USA) into rats who underwent vein grafting after transected nerve injury.
DM + curcumin group (DM + Cur)	In this group, 300 mg/kg/day of curcumin dissolved in olive oil was administered by oral gavage for 28 days for treatment to rats who underwent surgical procedures, as in the DM group
DM + *Garcinia kola* group (DM + GK)	In this group, 200 mg/kg/day of *Garcinia kola* dissolved in distilled water was administered by oral gavage for 7 days for treatment to rats who underwent surgical procedures as in the DM group.

**Table 4:** Antioxidant activity and phenolic content of *Garcinia kola*.

	Total Phenolic Content mg/g	DPPH, mmol/g Trolox Equivalent	FRAP, mmol/g Trolox Equivalent
*Garcinia kola*	59.41	136.98	213.515

## Data Availability

Data is contained within the article.

## Abbreviations

The following abbreviations are used in this manuscript:

2D	2-dimension
Cur	Curcumin
DM	Diabetes mellitus
GK	*Garcinia kola*
GFAP	Glial Fibrillary Acidic Protein
STZ	Streptozotocin
TNF-alpha	Tumor Necrosis Factor-alpha
TUNEL	Terminal deoxynucleotidyl transferase dUTP Nick End Labeling
